# Coronary Calcium Score for the Prediction of Asymptomatic Coronary Artery Disease in Patients With Ischemic Stroke

**DOI:** 10.3389/fneur.2020.00206

**Published:** 2020-03-27

**Authors:** Hye-Yeon Choi, Soo Jeong Shin, Joonsang Yoo, Kijeong Lee, Dongbeom Song, Young Dae Kim, Hyo Suk Nam, Kyung Yul Lee, Hye Sun Lee, Dong Joon Kim, Ji Hoe Heo

**Affiliations:** ^1^Department of Neurology, Kyung Hee University College of Medicine, Kyung Hee University Hospital at Gangdong, Seoul, South Korea; ^2^Department of Neurology, Yonsei University College of Medicine, Seoul, South Korea; ^3^Department of Neurology, Keimyung University School of Medicine, Daegu, South Korea; ^4^Department of Neurology, College of Medicine, Eunpyeong St. Mary Hospital, Catholic University of Korea, Seoul, South Korea; ^5^Biostatistics Collaboration Unit, Yonsei University College of Medicine, Seoul, South Korea; ^6^Department of Radiology, Yonsei University College of Medicine, Seoul, South Korea; ^7^Integrative Research Center for Cerebrovascular and Cardiovascular Diseases, Yonsei University College of Medicine, Yonsei University Health System, Seoul, South Korea

**Keywords:** cerebral infarction, coronary artery disease, coronary calcium score, atherosclerosis, risk factors

## Abstract

**Purpose:** Many patients with ischemic stroke have concomitant coronary artery disease (CAD). However, it remains unclear which stroke patients should undergo evaluation for asymptomatic CAD, and which screening tools are appropriate. We investigated the role of coronary artery calcium (CAC) score as a screening tool for asymptomatic but severe CAD in acute stroke patients. We determined the selection criteria for CAC screening based on risk factors and cerebral atherosclerosis.

**Materials and Methods:** The present study included consecutive patients with acute stroke who had undergone cerebral angiography and multi-detector computed tomography coronary angiography. Severe CAD was defined as left main artery disease or three-vessel disease. Enrolled patients were randomly assigned to two sets; a set for developing selection criteria and a set for validation. To develop selection criteria, we identified associated factors with severe CAD regarding clinical factors and cerebral atherosclerosis. CAD predictability of selection criteria with the CAC score was calculated.

**Results:** Overall, 2,658 patients were included. Severe CAD was present in 360 patients (13.5%). CAC score was associated with CAD severity (*P* < 0.001). In the development set (*N* = 1,860), severe CAD was associated with age >65 years [odds ratio (95% confidence interval), 2.62 (1.93–3.55)], male sex (1.81 [1.33–2.46]), dyslipidemia (1.77 [1.25–2.61]), peripheral artery disease (2.64 [1.37–5.06]) and stenosis in the cervicocephalic branches, including the internal carotid (2.79 [2.06–3.78]) and vertebrobasilar arteries (2.08 [1.57–2.76]). We determined the combination of clinical and arterial factors as the selection criteria for CAC evaluation. The cut-off criterion was two or more elements of the selection criteria. The area under the curve (AUC) of the selection criteria was 0.701. The AUC significantly improved to 0.836 when the CAC score was added (*P* < 0.001). In the validation set (*N* = 798), the AUC of the selection criteria only was 0.661, and that of the CAC score was 0.833. The AUC of the selection criteria + CAC score significantly improved to 0.861(*P* < 0.001).

**Conclusion:** The necessity for CAC evaluation could be determined based on the presence of risk factors and significant stenosis of the cervicocephalic arteries. CAC evaluation may be useful for screening for severe CAD in stroke patients.

## Introduction

Ischemic heart disease is the leading cause of long-term mortality in patients with stroke (1). The annual risk of myocardial infarction in patients with ischemic stroke is ~2.2% (1, 2). The presence and extent of asymptomatic stenosis in coronary angiography is strongly predictive of major cardiovascular events. Previous studies identified significant (≥50%) stenosis of the coronary artery in 20–41% of patients with stroke via autopsy, coronary angiography, or multi-detector computed tomography angiography (MDCTA) (3–7). Therefore, coronary screening may be necessary for stroke patients at high risk of coronary artery disease (CAD). However, it still remains uncertain which group of patients with stroke should undergo evaluation for asymptomatic CAD, and which evaluation tools are most appropriate for coronary screening in such patients.

Atherosclerosis is a systemic disease and CAD shares several risk factors with cerebral atherosclerosis (8). In fact, previous studies have demonstrated a significant association between CAD and atherosclerosis of the cervicocephalic arteries including the vertebrobasilar artery (VBA) and carotid arteries (4, 6, 9, 10). These findings suggest that CAD may be predicted to some extent by the presence of cerebral atherosclerosis and vascular risk factors.

Previous studies have indicated that coronary artery calcium (CAC) is superior to risk factor-based prediction of CAD and coronary events (11–14). Additionally, other studies reported that CAC scores are associated with the severity of CAD (15). In a large prospective population cohort registry, the risk of coronary events increased as the CAC score increased (12). This study aimed to investigate the role of the CAC score as a screening tool for the diagnosis of asymptomatic but severe CAD in patients with acute stroke. We also sought to determine the selection criteria for CAC screening in patients with stroke based on the presence of risk factors and cerebral atherosclerosis.

## Materials and Methods

### Study Population

Our study involved consecutive patients with acute ischemic stroke or transient ischemic attack who had been admitted to the hospital within seven days after the onset of symptoms. All admitted patients were evaluated using a standard protocol, which included brain computed tomography (CT), magnetic resonance imaging (MRI), cerebral angiographic studies (CT angiography, magnetic resonance (MR) angiography, or digital subtraction angiography), standard blood tests, 12-lead electrocardiography, continuous electrocardiography monitoring during hospitalization in the stroke unit or 24-h Holter monitoring, echocardiography, and MDCTA (16, 17). All patient data were entered into a prospective registry. Patients who had been admitted between January 2007 and August 2015 and had undergone both MDCTA and cerebral angiographic studies were included in the present study.

MDCTA was indicated when the patient fulfilled at least one of the following criteria: (1) presence of atherosclerosis in the intracranial or extracranial cerebral arteries; (2) presence of ≥2 risk factors for CAD such as hypertension, diabetes mellitus, dyslipidemia, cigarette smoking, or central obesity; and (3) when the patient's age is associated with increased risk of CAD (men >45 years; women >55 years). The exclusion criteria were as follows: (1) the presence of CAD (i.e., angiographically confirmed CAD, unstable angina, coronary artery stent, or angioplasty/coronary artery bypass graft) before index stroke; (2) high pulse rate (>65/min) that is not controlled using beta-blockers at the time of MDCTA; (3) poor cooperation; (4) impaired renal function (estimated glomerular filtration rate <60 mL/min/1.73 m^2^ or history of chronic kidney disease); and (5) failure to obtain informed consent (6, 18). Patients who were diagnosed to have significant CAD on MDCTA were consulted or referred to cardiologists for further evaluation and management. Some of them were treated with elective coronary revascularization.

The present study was approved by the Institutional Review Board of Severance Hospital, Yonsei University Health System (Seoul, South Korea). Informed consent for this study was waived due to the retrospective nature of the analysis.

### Assessment of CAC Score and Coronary Artery Disease

Patients underwent MDCTA within 2 weeks after admission, using a second-generation dual-source CT scanner (Somatom Definition Flash; Siemens Medical Solutions, Erlangen, Germany) with prospective electrocardiographic gating, 330-ms gantry rotation time, and a 120-kV tube. A beta-blocker (40 mg propranolol hydrochloride) was administered 1–2 h before the examination of patients with a resting pulse rate >65/min. Volume-rendered images, curved multiplanar images, and routine cardiac axis views were obtained using a dedicated workstation (Wizard; Siemens Medical Solutions) and interpreted by independent cardiac radiologists.

CAC was identified as a high-attenuation area (exceeding the threshold of 130 Hounsfield units in a minimum of three contiguous pixels) in the coronary artery. CAC scores were calculated according to the Agatston method (19) semi-automatically by cardiac radiologists during routine practice and were included in a formal report on coronary artery CT angiography. Patients were categorized into the following four subgroups based on their CAC scores: 0, 0.1–99.9, 100–399.9, and ≥400 (15, 20).

The presence of stenosis was determined at four main coronary branches: left main, left anterior descending, left circumflex, and right coronary arteries. The severity of obstructive CAD was primarily categorized as follows: no CAD, mild CAD (<50% stenosis), one-vessel disease, two-vessel disease, and three-vessel disease. One-, two-, or three-vessel disease is determined based on the number of arteries with significant stenosis (≥50%) among the three major coronary arteries (left anterior descending, left circumflex, and right coronary arteries). Severe CAD was defined as three-vessel disease or ≥50% stenosis in the left main artery based on the current treatment guideline for stable coronary artery disease (21).

### Assessment of Cerebral Atherosclerosis

Abnormalities observed during cerebral angiography were confirmed at a weekly stroke conference, based on a neuroradiologist's report and the consensus of stroke specialists. The degree of stenosis was evaluated using the North American Symptomatic Carotid Endarterectomy Trial and Warfarin vs. Aspirin for Symptomatic Intracranial Disease method (22, 23). The degree of stenosis was estimated in the carotid, vertebral, basilar, anterior cerebral, middle cerebral, and posterior cerebral arteries. When multiple stenotic lesions were present in one artery, data from the most severe lesion were used. Significant stenosis in cerebral artery atherosclerosis was defined as atherosclerotic stenosis ≥50% or an obstruction.

### Data Analysis and Statistics

Statistical significance was set at P <0.05. IBM SPSS Statistics for Windows (version 26.0, SPSS Inc., Chicago, IL, USA), R version 3.4.4, and MedCalc Statistical Software version 19.1.3 (MedCalc Software bvba, Ostend, Belgium; https://www.medcalc.org; 2019) were used for statistical analysis. All continuous data were expressed as mean ± standard deviation or median with interquartile range (IQR), and all categorical data were presented as numbers (percentages). Independent sample *t*-tests and Pearson χ^2^ tests were used to compare risk factors and the severity and location of cerebral artery atherosclerosis among the CAC score subgroups. Enrolled patients were randomly assigned to two different sets, a set for developing criteria and a set for validation.

#### Developing Selection Criteria for Performing CAC Scan

To determine which patients are at high risk of severe CAD, logistic regression analysis was performed. Odds ratios (ORs) with 95% confidence intervals (CIs) were calculated to estimate the risk associated with a particular variable, based on binomial distributions.

For the clinical factors, covariates included the presence of classic vascular risk factors and laboratory results. Statistically significant continuous variables in univariate logistic regression were transformed into binomial categorical variables using cut-off values deriving by ROC curve analysis.

For the arterial factors based on cerebral artery atherosclerosis, we used the presence of arterial stenosis as a covariate for univariate analysis. According to the results from univariate logistic regression analysis, we categorized cerebral arteries into two subgroups: the intracranial branches (middle cerebral artery [MCA], anterior cerebral artery [ACA], and posterior cerebral artery [PCA]) and cervicocephalic branches (internal carotid artery [ICA] and vertebrobasilar artery [VBA]). The number of arteries with significant stenosis in each group was examined in terms of the association with severe CAD for determining selection criteria for CAC screening.

Statistically significant variables resulting from multivariate logistic regression analysis were selected to determine the selection criteria for CAC screening. Finally, we developed simple selection criteria by combining the results of the analysis of clinical factors and cerebral atherosclerosis. The values of AUC of clinical factors, arterial factors, CAC score, and combined criteria were compared. Youden's J statistics using ROC curve analysis were used for determining the cut-off criterion of the selection criteria for performing a CAC scan.

#### Validating Selection Criteria

To test the efficacy of selection criteria, ROC curve analysis to predict severe CAD was done in the separate validation set.

## Results

### Study Population

Among the 5,745 patients admitted during the study period, 3,479 patients (60.6%) underwent MDCTA. After excluding patients without available CAC scores (780 patients) and those without cerebral angiographic data (41 patients), a total of 2,658 patients (46.2%) were finally included in the analysis. Enrolled patients were older and mostly male (*P* < 0.001). Hypertension, diabetes mellitus, dyslipidemia, metabolic syndrome, and peripheral artery disease were common among the enrolled patients (Supplementary Table 1), mainly attributable to the predetermined criteria for performing coronary CTA.

The mean age of the 2,658 patients enrolled in the study was 65 ± 11 years (range: 17–96). Among these patients, 1,745 patients were men (65.7%). Coronary artery disease with ≥50% stenosis in at least one coronary artery was detected in 1,382 patients (52%). Six hundred and fifty-eight patients (24.8%) had one-vessel disease, while 364 patients (13.7%) had two-vessel disease. Severe CAD (three-vessel disease or left main coronary artery disease) was present in 360 patients (13.5%). The median CAC score was 66.65 (IQR 3.40–282.25).

Enrolled patients were randomly assigned to two sets; a set for developing selection criteria for performing CAC scan (*N* = 1,860) and a set for testing its efficacy (*N* = 798) to predict severe CAD. There was no statistical difference between the two sub-population in terms of basic demographic factors, distribution of CAC scores, and frequency of cardiovascular risk factors, and mean laboratory values (Table 1).

**Table 1:** Demographic differences between the set for developing criteria and the set for validation.

	**The set for developing selection criteria (*N* = 1,860)**	**The set for validation (*N* = 798)**	***p***
Age	65.5 ± 11.1	64.7 ± 11.2	0.078
Sex, male	1214 (65.3)	531 (66.5)	0.568
CAC score*	70.55 (3.05-293.45)	56.55 (3.68-245.40)	0.099
CAC group			0.409
CAC score 0	346 (18.6)	139 (17.4)	
0 < CAC score <100	698 (37.5)	327 (41.0)	
100 ≤ CAC score <400	457 (24.6)	183 (22.9)	
CAC score ≥ 400	359 (19.3)	149 (18.7)	
Severe coronary artery disease	256 (13.8)	104 (13.0)	0.658
Risk factors			
Hypertension	1416 (76.1)	607 (76.1)	1
Diabetes mellitus	612 (32.9)	262 (32.8)	1
Dyslipidemia	247 (13.3)	92 (11.5)	0.239
Smoking	491 (26.4)	234 (29.3)	0.132
Metabolic syndrome	786 (42.3)	347 (43.5)	0.587
Atrial fibrillation	242 (13.0)	97 (12.2)	0.587
Laboratory findings			
Total cholesterol	183.4 ± 61.5	182.7 ± 64.9	0.807
Triglyceride	129.1 ± 95.0	127.2 ± 84.2	0.618
HDL cholesterol	42.3 ± 10.6	42.2 ± 11.1	0.759
LDL cholesterol	108.7 ± 35.4	107.9 ± 35.8	0.631
HbA1c	6.7 ± 3.5	6.5 ± 1.4	0.051
CRP	13.2 ± 32.0	11.5 ± 29.8	0.442
hs-CRP	6.9 ± 18.8	6.6 ± 21.6	0.741
Initial glucose	142.7 ± 62.5	140.7 ± 56.3	0.421
Fasting glucose	115.6 ± 41.5	116.9 ± 70.1	0.621
Initial systolic BP	155.7 ± 39.7	155.6 ± 31.0	0.903
Initial diastolic BP	85.6 ± 15.5	86.6 ± 16.3	0.161
Previous cerebral infarction	250 (13.4)	113 (14.2)	0.665
Peripheral artery disease	45 (2.4)	13 (1.6)	0.257

Values are presented as the mean ± standard deviation or number (%), except CAC score*.

CAC scores* are presented as the median (interquartile range).

*BP, blood pressure; CAC, coronary artery calcium; HbA1c, hemoglobin A1c; HDL, high-density lipoprotein; hs-CRP, high-sensitivity C-reactive protein; LDL, low-density lipoprotein*.

### Developing Selection Criteria

#### Predicting Severe Coronary Artery Disease Using the CAC Score

**Figure 1:**
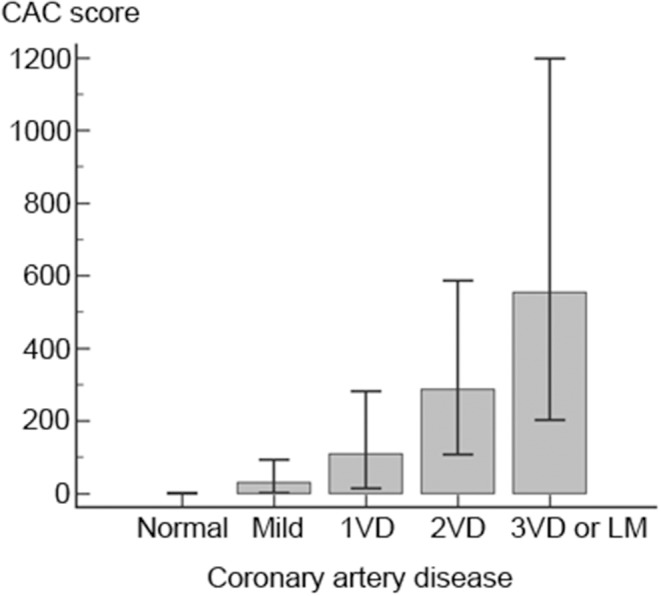
Coronary calcium score related to the severity of coronary artery disease. Coronary calcium scores (CAC) significantly increased as coronary artery severity increased. CAC was shown as the median and interquartile ranges. CAC, coronary artery calcium; LM, left main; VD, vessel disease.

Among 1,860 patients in the set for developing selection criteria, coronary artery disease with ≥50% stenosis in at least one coronary artery was detected in 983 patients (52.8%). Severe CAD (three-vessel disease or left main coronary artery disease) was present in 256 patients (13.8%). The CAC score was significantly higher in the patients with severe CAD (median 554.4, IQR 202.7–1198.0) compared with the patients without severe CAD (median 48.2, IQR 1.0–195.2, *P* < 0.001). The CAC score increased as CAD severity (Figure 1). The CAC score of each subgroup was significantly correlated with the severe CAD and the strength of this correlation increased as the CAC score increased (Table 2).

**Table 2:** Coronary artery calcium score correlated with severe coronary artery disease.

	**Odds ratio (95% CI)**	***P***
CAC group		
CAC score 0	Reference	
0 < CAC score <100	6.77 (2.08–22.05)	0.002
100 ≤ CAC score <400	16.95 (5.27–54.55)	<0.0001
CAC score ≥ 400	86.87 (27.35–275.88)	<0.0001

*CAC, coronary artery calcium; CI, confidence interval*.

#### Factors Associated With Severe CAD

##### Classic vascular risk factors and laboratory results

In univariate logistic regression analysis, severe CAD was associated with old age, male sex, dyslipidemia, peripheral artery disease, previous history of cerebral infarction, high levels of fasting glucose, and low levels of high-density lipoprotein cholesterol (Table 3). The age of 65 years was the cut-off for predicting severe CAD in the ROC curve analysis. Severe CAD was independently associated with age, male sex, dyslipidemia, and peripheral artery disease in the multivariate logistic regression analysis (Table 3). These clinical factors (including age >65 years, the male sex, dyslipidemia, and peripheral artery disease) were used to develop the selection criteria for performing a CAC scan to screen severe CAD.

**Table 3:** Association between severe coronary artery disease and clinical factors.

	**Univariate analysis**		Multivariate analysis^*^		**Multivariate analysis^†^**	
	**Odds ratio (95% CI)**	***P***	**Odds ratio (95% CI)**	***P***	**Odds ratio (95% CI)**	***P***
Age	1.05 (1.04–1.06)	<0.0001	1.06 (1.04 −1.07)	<0.0001		
Age (>65)	2.45 (1.83–3.28)	<0.0001			2.62 (1.93–3.55)	<0.0001
Sex (male)	1.59 (1.18–2.14)	0.002	1.95 (1.42–2.67)	<0.0001	1.81 (1.33–2.46)	<0.0001
Risk factors						
Hypertension	1.6 (1.14–2.26)	0.007	1.32 (0.92–1.88)	0.134	1.40 (0.98–2.00)	0.062
Diabetes mellitus	1.39 (1.06–1.83)	0.017	1.09 (0.78–1.50)	0.623	1.21 (0.89–1.66)	0.232
Dyslipidemia	1.68 (1.19–2.37)	0.003	1.81 (1.25–2.61)	0.002	1.77 (1.25–2.61)	0.002
Smoking	1.11 (0.82–1.49)	0.5				
Metabolic syndrome	0.96 (0.74–1.26)	0.766				
Peripheral artery disease	3.62 (1.94–6.77)	<0.0001	2.32 (1.19–4.51)	0.014	2.64 (1.37–5.06)	0.004
Previous cerebral infarction	1.6 (1.13–2.27)	0.008	1.39 (0.97–2.00)	0.073	1.32 (0.92–2.53)	0.133
Laboratory findings						
HbA1c	1.00 (0.95–1.04)	0.845				
Initial glucose	1.00 (1.00–1.00)	0.137				
Fasting glucose	1.00 (1.00–1.00)	0.008	1.01 (1.00–1.01)	0.008		
Fasting glucose (>113mg/dl)	1.32 (1.01–1.73)	0.04			1.19 (0.88–1.62)	0.26
C-reactive protein	1.01 (1.00–1.01)	0.296				
hs-CRP	1.00 (1.00–1.01)	0.479				
Total cholesterol	1.00 (1.00–1.00)	0.703				
Triglyceride	1.00 (1.00–1.00)	0.941				
HDL cholesterol	0.99 (0.97–1.00)	0.029	0.99 (0.98–1.01)	0.331		
HDL cholesterol (≤ 41mg/dl)	1.34 (1.03–1.75)	0.031			1.23 (0.93–1.62)	0.144
LDL cholesterol	1.00 (1.00–1.01)	0.427				

* *Was performed with age, levels of laboratory findings as continuous covariates*.

† *Was performed with age, fasting glucose, and HDL cholesterol as categorical covariates*.

*CI, confidence interval; HbA1c, hemoglobin A1c; HDL, high-density lipoprotein; hs-CRP, high-sensitivity C-reactive protein; LDL, low-density lipoprotein*.

##### Cerebral atherosclerosis

Various degrees of cerebral atherosclerotic stenosis was detected in 1,389 patients (74.7%) among 1,860 patients. Cerebral atherosclerosis with significant (≥50%) stenosis at different locations was detected in 1,021 patients (54.9%). Severe CAD was significantly associated with significant (≥50%) stenosis in the BA, VA, and ICA (Table 4). To simplify the criteria, the stenotic lesion in the VA and BA were combined as VBA.

**Table 4:** Association between cerebral atherosclerosis and severe coronary artery disease.

		**Univariate analysis**		Multivariate analysis^*^		**Multivariate analysis^†^**	
		**Odds ratio (95% CI)**	***P***	**Odds ratio (95% CI)**	***P***	**Odds ratio (95% CI)**	***P***
ACA	<50	Reference					
	≥50	1.69 (0.94–3.03)	0.079				
MCA	<50	Reference					
	≥50	1.33 (0.898–1.80)	0.07				
PCA	<50	Reference					
	≥50	1.41(0.96–2.05)	0.078				
ICA	<50	Reference		Reference		Reference	
	≥50	3.05 (2.26–4.11)	<0.0001	2.87 (2.12–3.88)	<0.0001	2.79 (2.06–3.78)	<0.0001
BA	<50	Reference		Reference			
	≥50	1.96 (1.15–3.34)	0.013	1.80 (1.05–3.09)	0.033		
VA	<50	Reference		Reference			
	≥50	2.11 (1.58–2.81)	<0.0001	1.90 (1.41–2.54)	<0.0001		
VBA	<50	Reference				Reference	
	≥50	2.30 (1.74–3.04)	<0.0001			2.08 (1.57–2.76)	<0.0001
Number of arteries with significant stenosis							
Intracranial branches	0	Reference					
	1	1.41 (1.06–1.88)	0.019				
	2	1.52 (0.82–2.83)	0.187				
	3	3.06 (0.78–11.94)	0.108				
Cervicocephalic branches	0	Reference					
	1	2.31 (1.74–3.08)	<0.0001				
	2	5.43 (3.42–8.61)	<0.0001				

* *was performed with BA and VA as separate covariates*.

† *was performed with VBA as a covariate*.

*ACA, anterior cerebral artery; BA, basilar artery; CAC, coronary artery calcium; CI, confidence interval; ICA, internal carotid artery; MCA, middle cerebral artery; PCA, posterior cerebral artery; VA, vertebral artery; VBA, vertebrobasilar artery*.

The association between stenosis of cervicocephalic branches (VBA and ICA) and severe CAD was significant and strengthened as the number of arteries with stenosis increased. However, there was no significant association between stenosis of intracranial branches and severe CAD (Table 4). The number of cervicocephalic branches with significant stenosis was determined as the arterial factors of the selection criteria for performing a CAC scan.

#### Determination of Selection Criteria for Performing CAC Scan

**Figure 2:**
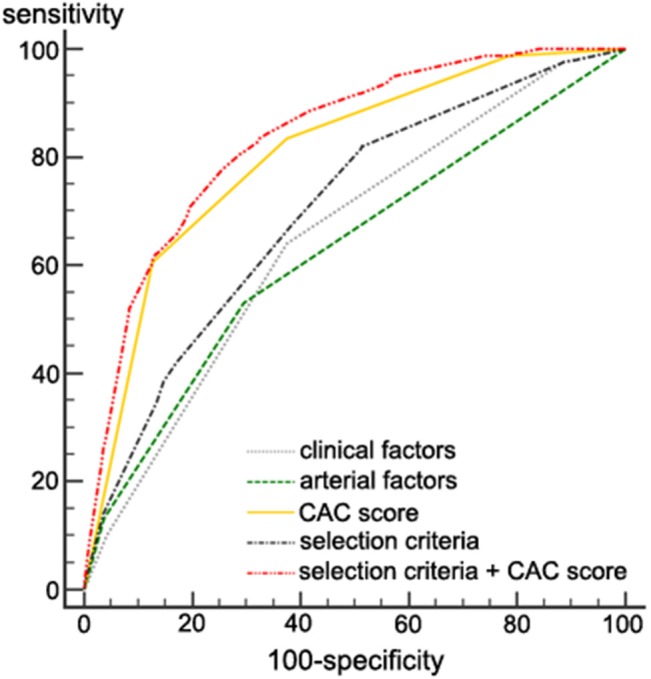
Severe coronary artery disease predictability in the development set. The AUC value of the selection criteria ± the CAC score for predicting severe CAD was 0.836. It was significantly higher than that of a CAC score alone (0.805), clinical factors only (0.652), arterial factors only (0.628), and the selection criteria (a combination of clinical and arterial factors, 0.701). *P* < 0.001 for all comparisons. AUC, area under the curve; CAC, coronary artery calcium; CAD, coronary artery disease.

We developed criteria to select which stroke patients need to perform CAC scan based on the results determining clinical and arterial factors of selection criteria. We included age, sex, and other variables that were significant in multivariate analyses to determine elements of the selection criteria combining clinical and arterial factors (Tables 3, 4). We calculated the odds ratio of each potential component of the selection criteria combining clinical and arterial factors for severe CAD (Table 5). The final selection criteria combining clinical and arterial factors were age (>65), male sex, dyslipidemia, peripheral artery disease, and the presence of significant stenosis in the internal carotid artery or the vertebrobasilar artery. The AUC of selection criteria (a combination of clinical and arterial factors) for predicting severe CAD was 0.701, which significantly higher than that of clinical factors only (0.652) and that of arterial factors (0.628) (*P* < 0.001). The AUC of the CAC score was 0.805. The predictability significantly improved to 0.836 when the CAC score was added to the selection criteria (*P* < 0.001, Figure 2).

**Table 5:** Adjusted Odds ratio with selected factors for developing selection criteria.

	**Odds ratio (95% CI)**	***P***
Age (>65)	2.36 (1.74–3.20)	<0.0001
Sex (male)	1.66 (1.22–2.27)	0.001
Dyslipidemia	1.73 (1.21–2.50)	0.003
Peripheral artery disease	2.76 (1.41–5.41)	0.003
ICA stenosis (≥50%)	2.42 (1.77–3.30)	<0.0001
VBA stenosis (≥50%)	1.92 (1.44–2.57)	<0.0001

*CI, confidence interval; ICA, internal carotid artery; VBA, vertebrobasilar artery*.

The cut-off criterion of the selection criteria for CAC screening was determined as two or more elements among the selection criteria (a combination of clinical and arterial factors) based on Youden's J statistics using ROC curve analysis (Supplementary Table 2). The sensitivity was 82.03 and the specificity was 48.50. A CAC score ≥100 was the predicting cut-off value for severe CAD by ROC curve analysis.

### Testing Selection Criteria for Performing CAC Scan

**Figure 3:**
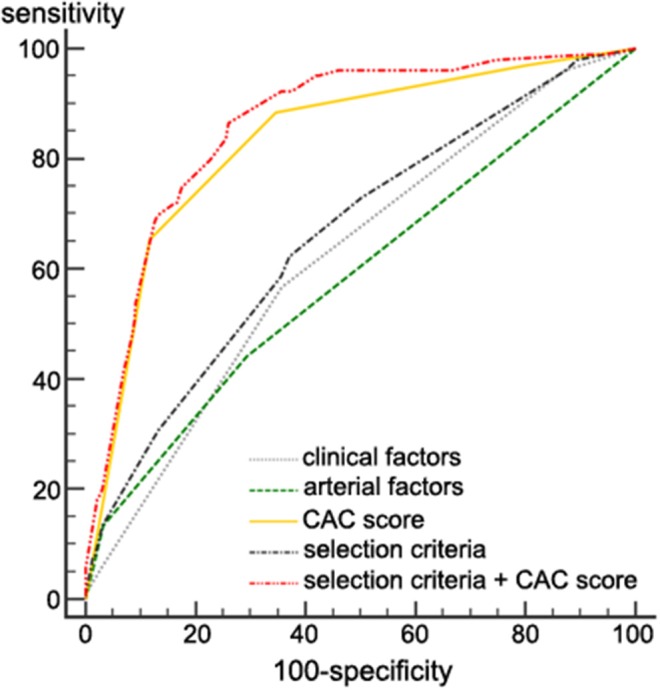
Severe coronary artery disease predictability in the validation set. The AUC value of the selection criteria ± the CAC score for predicting severe CAD was 0.860. It was significantly higher than that of a CAC score alone (0.833), clinical factors only (0.623), arterial factors only (0.587), and the selection criteria (a combination of clinical and arterial factors, 0.661). *P* < 0.001 for all comparisons. AUC, area under the curve; CAC, coronary artery calcium; CAD, coronary artery disease.

In the validation set, the AUC value of selection criteria for predicting severe CAD was 0.661 (Figure 3). The AUC value of the selection criteria with a CAC score was 0.860 (Figure 3). It was higher than the predictability of a CAC score alone (the AUC value 0.833) or selection criteria alone (0.661, *P* < 0.001).

## Discussion

In this study, we first showed that the CAC score was correlated with CAD severity. We then demonstrated that severe CAD was associated with clinical factors (age >65 years, male sex, dyslipidemia, and peripheral artery disease) and arterial factors (presence of significant stenosis in cervicocephalic branches (ICA or VBA). Finally, we determined the optimal selection criteria for CAC evaluation in stroke patients, which was a combination of clinical and arterial factors. The cut-off criterion of the selection criteria were two or more factors of the selection criteria. Furthermore, a CAC score ≥100 predicted severe CAD in patients with acute stroke in the development set. In the validation set, severe CAD predictability of the selection criteria with a CAC score was higher than a CAC score alone.

Current guidelines do not recommend screening tests for the evaluation of CAD in asymptomatic populations (24). However, evidence regarding this in stroke patients is still scarce. Among stroke patients with asymptomatic CAD, major vascular events are frequent in those who are diagnosed with CAD via coronary angiography (7). In a study involving 1,893 patients with acute ischemic stroke, asymptomatic CAD detected via coronary MDCTA was associated with an increased risk of vascular events or death during long-term follow-up (18). Propensity score matching analyses in patients with acute ischemic stroke showed that vascular events and death were less frequent among those who had undergone MDCTA than in those who had not during the follow-up period (25). These findings suggest that screening to evaluate asymptomatic CAD may be beneficial in a subgroup of patients with acute cerebral infarction.

The Framingham risk score is commonly used to predict CAD (26, 27). It is a multivariable statistical model that uses a risk factor-based paradigm, which includes variables such as age, sex, smoking, total cholesterol, LDL cholesterol, systolic blood pressure, and pharmacological treatments for high blood pressure. The current American Heart Association guideline recommends the use of the pooled cohort equations to assess the 10-years atherosclerotic cardiovascular disease risk (28).

The Predicting Asymptomatic Coronary Artery Disease in Patients with Ischemic Stroke and Transient Ischemic Attack (PRECORIS) score considers the presence and severity of cervicocephalic arterial stenosis, in addition to the Framingham risk score. The PRECORIS score was useful for detecting CAD in patients with stroke (4, 29). We also showed that the risk of CAD was high in stroke patients with multiple risk factors in addition to the presence of atherosclerosis of the carotid and/or vertebrobasilar arteries (6). These findings suggest that the consideration of both the risk factors and the presence of cervicocephalic arterial stenosis helps to predict CAD in patients with stroke better. Patients with acute stroke routinely undergo cerebral angiography. Therefore, information on the stenosis of cervicocephalic arteries can be easily obtained. In the present study, we showed that the use of simple criteria based on the number of risk factors and the significance of stenosis of the cervicocephalic arteries could help to determine whether CAC screening is required or not. These criteria may be useful in daily clinical practice.

Cardiac CTA is a sensitive and specific diagnostic tool for CAD. However, it is not recommended for screening asymptomatic CAD because of the potentially harmful side effects (30). Cardiac CTA requires the administration of contrast agents, which limits its use in patients with chronic kidney disease. These contrast agents have been associated with idiosyncratic side effects. Beta-blockers are also often required for heart rate control. CAC scores obtained via non-contrast cardiac CT have been widely utilized for the prediction of ischemic heart disease (30). Although coronary CTA is superior to CAC for the prediction of major cardiovascular events (13), CAC measurement is non-invasive, does not require the use of contrast agents, and can be performed very rapidly and efficiently.

The present study has some limitations. First, CAC scores in our study population may have been higher than those in the general population, since all enrolled patients had experienced a stroke or transient ischemic attack and had risk factors that indicate the necessity for coronary CTA. Second, this study was based on data obtained from a single hospital-based registry, in which all patients were of the same ethnicity. Since the prevalence and risk of CAD and cerebral atherosclerosis may differ among patients with different ethnicities and those in different countries (25), caution should be applied when generalizing our results to other populations. The results are conditional on subjects prejudged to be at high risk for atherosclerotic coronary events by virtue of the presence of atherosclerosis in intra- or extracranial cerebral arteries. Further studies are required to validate our findings in other study populations. Third, potential selection bias presents in this study because MDCTA was indicated in patients with high risks of CAD.

In conclusion, our findings suggest that the necessity for CAC evaluation can be determined based on the presence of clinical risk factors and significant stenosis of the cervicocephalic arteries in acute ischemic stroke patients. CAC evaluation may be useful for predicting the severity of CAD in patients with ischemic stroke.

## Data Availability Statement

The datasets generated for this study are available on request to the corresponding author.

## Ethics Statement

The studies involving human participants were reviewed and approved by Institutional Review Board of Severance Hospital, Yonsei University Health System. Written informed consent for participation was not required for this study in accordance with the national legislation and the institutional requirements.

## Author Contributions

H-YC, SJS, and JHH contributed to the conception and design of the study. YDK, HSN, JY, KL, and DS organized the database. H-YC and HSL performed the statistical analysis. H-YC wrote the first draft of the manuscript. DJK and KYL provided critical review. JHH reviewed, edited, and revised the manuscript. All authors contributed to manuscript revision, read, and approved the submitted version.

### Conflict of Interest

The authors declare that the research was conducted in the absence of any commercial or financial relationships that could be construed as a potential conflict of interest.
