# Supplementary material for: Coronary Calcium Score for the Prediction of Asymptomatic Coronary Artery Disease in Patients With Ischemic Stroke
Source: Front Neurol. 2020 Mar 27;11:206. doi: 10.3389/fneur.2020.00206 (PMC7134382; doi:10.3389/fneur.2020.00206)
Supplement: Supplementary file 1 [file Data_Sheet_1.pdf]

Supplement Table 1. Demographic characteristics of excluded and enrolled patients

	Excluded patients (N=3087)	Enrolled patients (N=2658)	p
Age	66.4 ± 13.9	65.3 ± 11.2	0.001
Male	1719 (55.8)	1745 (65.7)	<0.0001
Hypertension	2226 (72.1)	2023 (76.1)	0.001
Diabetes Mellitus	952 (30.8)	874 (32.9)	0.103
Dyslipidemia	325 (10.5)	339 (12.8)	0.01
Smoking	544 (17.6)	725 (27.3)	<0.0001
Valvular heart disease	218 (7.1)	106 (4.0)	<0.0001
Atrial fibrillation	725 (23.5)	339 (12.8)	<0.0001
Previous cerebral infarction	624 (20.2)	363 (13.7)	<0.0001
Peripheral artery disease	123 (4.0)	58 (2.2)	<0.0001
Metabolic syndrome	1193 (38.6)	1133 (42.6)	0.002
Initial systolic BP	156.1 ± 285.1	155.7 ± 37.3	0.933
Initial diastolic BP	83.5 ± 16.5	85.9 ± 15.8	<0.0001
Laboratory findings			
ESR	26.3 ± 24.0	21.1 ± 20.9	<0.0001
Fibrinogen	331.3 ± 224.9	309.9 ± 102.0	<0.0001
Initial glucose	142.6 ± 66.3	142.1 ± 60.7	0.773
HbA1c	6.7 ± 4.1	6.7 ± 3.0	0.968
Total cholesterol	170.3 ± 44.9	183.2 ± 62.6	<0.0001
Triglyceride	115.9 ± 76.8	128.5 ± 91.9	<0.0001
LDL cholesterol	102.2 ± 41.6	108.4 ± 35.5	<0.0001
HDL cholesterol	42.9 ± 12.3	42.3 ± 10.8	0.061

Values are presented as mean ± standard deviation or number (%).
BP, Blood pressure; ESR, erythrocyte sedimentation rate; HbA1c, hemoglobin A1c; LDL, low density lipoprotein; HDL, high density lipoprotein

Supplement Table 2. Sensitivity and specificity of each cut-off point of the selection criteria for performing CAC scan to predict severe coronary artery disease.

Criterion	Sensitivity	95% CI	Specificity	95% CI
>0	98.05	95.5 - 99.4	9.23	7.9 - 10.8
>1	82.03	76.8 - 86.5	48.5	46.0 - 51.0
>2	42.58	36.4 - 48.9	82.73	80.8 - 84.6
>3	14.45	10.4 - 19.4	96.2	95.1 - 97.1
>4	1.56	0.4 - 4.0	99.63	99.2 - 99.9
>5	0	0.0 - 1.4	99.94	99.7 - 100.0

CI, confidence interval
